# Psychometric Properties of the Arabic NEO-FFI-3 among Undergraduates in Kuwait

**DOI:** 10.1192/j.eurpsy.2025.1903

**Published:** 2025-08-26

**Authors:** B. M. Alansari

**Affiliations:** 1 Psychology, Kuwait University, Shuwaikh Campus, Kuwait

## Abstract

**Introduction:**

The reproduction of the structure of the NEO-FFI has been stable across different Arabic cultures. The NEO Five-Factor Inventory-3 (NEO-FFI-3) is the revised version of the NEO-FFI-R, which describes personality in terms of the Five Factor Model, namely Neuroticism, Extraversion, Openness to Experience, Agreeableness, and Conscientiousness. The psychometric properties of the NEO-FFI-3 present a robust verification base in diverse cultures. Although there is an Arabic version of the NEO-FFI, the psychometric properties of the Arabic NEO-FFI-3 are yet unknown.

**Objectives:**

The study aims to investigate the psychometric properties of the Arabic adaptation of the NEO-FFI-3.

**Methods:**

The Arabic version of the NEO-FFI-3 is a 60-item questionnaire, and the NEO-FFI-R 60-item questionnaire was administered to1373 Kuwait university undergraduates (559 males mean age = 20.41±1.43 and 814 females; mean age = 20.60±1.18). The internal consistency reliability, factor structure, and convergent validity of the NEO-FFI-3 with NEO–FFI-R were assessed.

**Results:**

Cronbach’s alpha was satisfactory for N (0.72), E (0.82), O (0.79), A (0.82) and C (0.74). Results revealed significant gender differences in N, O & C with a favor for females. PCA showed that NEO-FFI-3 five factors explains 53.98% of the total variance. However, the high mean correlations between the NEO-FFI-3 and NEO–FFI-R scales, with coefficients of (0.87) for the N, (0.85) for the E, (0.84) for the C, (0.78) for the O, and (0.77) for the A.

**Conclusions:**

The findings support the psychometric properties of the Arabic adaptations of the NEO-FFI-3 as useful instruments for assessing the Big Five.

**Disclosure of Interest:**

None Declared

